# Thiazole derivative based topical nanoemulgel for inhibition of bacterial virulence in surface infections 

**DOI:** 10.22038/IJBMS.2022.59419.13192

**Published:** 2022-03

**Authors:** Snigdha Bhardwaj, Sonam Bhatia, Pushpraj S. Gupta, Shaminder Singh

**Affiliations:** 1 Department of Pharmaceutical Science, SHALOM Institute of Health and Allied Sciences, Sam Higginbottom University of Agriculture,; 2 Technology and Sciences (SHUATS), Naini, Prayagraj, India; 3 I.T.S College of Pharmacy, Murad Nagar, Ghaziabad, U.P. (201206), India; 4 Regional Centre for Biotechnology, Department of Biotechnology (DBT), Faridabad, Haryana, 121001, India. 411040; # These authors contributed equally to this work

**Keywords:** Anti-virulent strategy, Drug-resistance, LasR, Nanoemulgel formulation, Pseudomonas aeruginosa, Thiazole compound

## Abstract

**Objective(s):**

Antimicrobial resistance emerged as a global challenge owing to limited therapeutic options to control infections. *Pseudomonas aeruginosa*, an MDR pathogen already developed resistance against many conventional antibiotics. An “anti-virulence strategy” that targets bacterial virulence rather than growth proves effective against drug-resistant pathogens.

**Materials and Methods:**

Here, we used a structure-based drug design approach to identify lead molecules using the LasR receptor protein of *P. aeruginosa* as a target responsible for virulence production in this bacterium. From the identified hits, we developed lead-based nanoformulation and investigated its effectiveness for treating the *P. aeruginosa* associated surface-infection *in-vivo*. First, TC-based nanoemulsions were fabricated by high-pressure homogenization and evaluated for various *in vitro* parameters. The optimized nanoemulsions were thereby utilized to prepare NEG.

**Results:**

The nanoemulsion (F3) exhibited low droplet size (51.04±1.88 nm), PDI (0.065±1.14), and negative zeta potential (-33.65±0.82 mV). In animals, topical application of NEG-3 demonstrated significant improvement on skin permeability (459±10.17 µg), drug influx (18.99±0.76 μg/cm^2^ hr), and repressed the CFU of *P. aeruginosa* induced-surface infection (*P*≤ 0.001). The histology of rat skin demonstrated a significant effect for groups treated with TC-based NEGs as compared with a negative control group, whereas no significant effect was seen on rat liver indicating low systemic exposure to the drug. Also, NEG3 showed no significant changes under different stability conditions after 3 months.

**Conclusion:**

TC-based NEGs open up the possibility of a more effective way to combat serious surface infections caused by *P. aeruginosa.*

## Introduction

**Figure 1:**
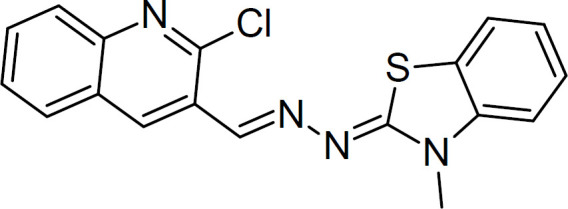
Chemical structure of thiazole compound (C_18_H_13_ClN_4_S) (2-((2-Chloroquinolin-3-yl)methylene)hydrazono)-3-methyl-2,3dihydrobenzo[d]thiaz)

Over the past decades, the reports on bacterial-resistant infections have increased outrageously. The pathogenic community is becoming resistant to available antimicrobial agents making the treatment regime ineffective and resulting in deadly infections. A dire need is emerging to develop alternative strategies to suppress infections caused by multi-drug resistant pathogens. *Pseudomonas aeruginosa*, a multidrug-resistant strain, frequently seen to be associated with healthcare infections, is more difficult to eradicate (1, 2). Nowadays, targeting virulence traits of infectious agents is an alternative approach to antimicrobials that are gaining much popularity to fight antimicrobial resistance. Bacteria utilize quorum sensing (QS) mechanism (communication process between bacteria) and govern the expression of various bacterial genes involved in regulating specific processes like biofilm formation, expression of virulence factors, production of secondary metabolites, bacterial secretory system, etc., as a result bacteria become resistant towards various antimicrobial agents. A novel anti-virulence strategy focuses on the inhibition of virulent expression by targeting the QS process without affecting the growth of pathogens (3-5). This non-killing approach renders the resistance rate low as the survival of pathogens does not affect the active drug, resulting in inhibition of bacterial pathogenicity. QS circuit of *P. aeruginosa *comprises various signaling pathways such as LasR, RhlR, Pqs, and QScR transcriptional proteins, and targeting this circuit will help in blocking downstream signal transducers which could result in suppressing bacterial virulence (6, 7). Chourasiya *et al*. (2015) performed molecular docking analysis of thiazole-based hit compound (C_18_H_13_ClN_4_S) (Figure 1) to examine its potential to bind to LasR protein of *P. aeruginosa. *The study revealed strong pieces of evidence in support of thiazole compound (TC) suppression of virulence of *P. aeruginosa *by blocking the LasR gene (a quorum-sensing signaling receptor), suggesting it as a potent QS inhibitor of *P. aeruginosa*. This study opens up scope for further development of TC-based formulations to curb the pathogenesis of *P. aeruginosa* infections (8). 

Recently, nanomaterials have emerged as efficient tools to deliver lipophilic bioactive. Various reports have been published suggesting nanoemulgel to be the most effective delivery system for topical application of drugs with high lipophilicity and low systemic availability or both. Unlike, conventional systems, nanoemulgel exhibited better partitioning of the drug through a percutaneous membrane at the target site and also improves pharmacokinetic behavior of the drug candidate with respect to solubility, permeability, non-greasy nature, non-toxicity, avoiding hepatic first-pass metabolism, safety, and bioavailability via topical route (9-14). The system shows high mucoadhesion, thus acts as a drug reservoir for sustained release of drug and improved patient compliance. The selection of appropriate vehicles, surfactants/co-surfactants, and methods of fabrication has a huge impact on stability and biological behavior of the developed system. The choice of surfactant is of important concern as the increased concentration of surfactants may cause mild to severe toxicity (15, 16). The research work carried out in this study is the continuation of work reported by Chourasiya *et al*., (2015) (8). Therefore, the study aimed at developing a TC-based nanoemulgel formulation and investigating its inhibitory effect against *P. aeruginosa* induced surface infection *in vivo*. 

## Materials and Methods

***Materials***

Thiazole compound (2-((2-Chloroquinolin-3-yl)methylene)hydrazono)-3-methyl-2,3dihydrobenzo[d]thiaz) (Figure 1) was purchased from Hebei Zhuangkai Biotechnology Ltd., China. Labrafac Lipophile, Capryol 90, Captex 355, Transcutol HP were purchased from Gattefosse, Mumbai, India. Tween-20, PEG-400, carbopol 934, and carbopol 940 were purchased from CDH, India. All chemicals used in the experimentation were of analytical grade. *P. aeruginosa* bacterial strain (PAO1) used in this study was gifted by D. Shaminder Singh (Ex-Assistant Professor of JIBB, SHUATS). 

***Methods***

***Selection of excipients***

The drug must show high solubility in the oil phase and the hydrophilic-lipophilic balance (HLB) value of surfactants should be higher than 10 to achieve a stable nanoemulsion. Also, the mixture of surfactant and co-surfactants with high and low HLB values may be used to maintain stable nanoemulsion (17, 18).

*Solubility studies in oil and surfactants*

The solubility of TC was analyzed by the shake flask method in different oil phases and surfactants. An aliquot amount of TC was added into 2 ml of oil and surfactant components to be tested, then water was mixed by using a vortex mixer on temperature maintained at 25±1 °C. The vials containing the drug sample were then kept in an isothermal shaker for 48 hr to maintain equilibrium. Afterward, the sample was subjected to centrifugation (4000 rpm) for 15 min and the above layer was filtered by a membrane filter (0.45 µm). The amount of TC dissolved (concentration) in varying supernatants was analyzed by using UV spectroscopy at 306 nm. Surfactant, co-surfactant, and oil phases were used based on the drug’s solubility in the same and were used further for nanoemulsion preparation (18). 

*Screening and optimization of the formulation components*

The phase solubility of TC in the oil phase, surfactant, and co-surfactant mixture (S-CoS mix) was investigated via a shake-flask method. The phases were selected based on the maximum solubility shown by TC in oil components. The different surfactants and co-surfactants were shortlisted based on emulsification efficiency for the oil phase as well as maximum solubility shown by a drug. The co-surfactant was selected based on the respective maximum nano-emulsifying areas observed in the phase diagram for each S-CoS optimized oil and surfactant component. (17, 19).

*Development of pseudo-ternary phase diagram*

The phase behavior of a mixture of oil and surfactants used in the nanoemulsion system was analyzed through interpretation of a phase diagram and constructed on pseudo-ternary plots using the aqueous titration method. The percent proportion of the oil phase, S-CoS mix, and the aqueous phase was determined and the phase diagram was constructed. S-CoS mixes were prepared by using different ratios such as 1:1, 2:1, and 3:2. For each phase plot, the oil and S-CoS mix ratios were mixed thoroughly in different volume ratios from 1:9 to 9:1 in different glass vials. Slow titration with aqueous phase (amount ranged between 5 % to 95%) was done separately for each combination of oil and S-CoS mix. (20, 21).

***Preparation of nanoemulsion***

In total three water in oil (w/o) types of nanoemulsion formulations (F1, F2, F3) were prepared. The amount of TC used in nanoemulsion formulations (F1, F2, and F3) was 0.05, 0.075, and 0.1 mg, respectively. The accurately weighed amount of TC was dissolved in the optimized homogenous oil phase (Labrafac Lipophile and Capryol 90 in 2:1 ratio) and S-CoS mix (Tween 20-Transcutol HP in a ratio of 1:1, 2:1, and 3:2) by using the homogenization process with the gradual addition of aqueous phase. The prepared emulsion was introduced in ultrasonic homogenization for 15 min (with on/off of 30 sec pulse) using an ultrasonic homogenizer (Sonics-Vibracell, USA) to obtain a transparent dispersion system. The nanoemulsion was placed in a beaker containing ice to neutralize heat generated during the process. The prepared nanoemulsion was further used for nanoemulgel formulation (22). 

***Characterization of nanoemulsions***

*Physical appearance*

All three developed nanoemulsion formulations (F1-F3) were analyzed manually for physical appearance as well as emulsion instability parameters such as creaming, cracking, and coalescence. 

*Drug content determination*

Accurately weighed quantity of nanoemulsion formulation (10 ml) was diluted with methanol (extracting solvent) and sonicated for 30 min. After sonication, the extract was centrifuged at 4000 rpm for 15 min. The supernatant layer was removed using a membrane filter (0.45 μm pore size). The dilutions were made with phosphate buffer saline (PBS) of pH 7.4 and the TC content was analyzed using UV spectroscopy at 306 nm. The examination was performed in triplicate and mean percent values were taken for final calculations (23). 

*Mean droplet size and polydispersity index (PDI)*

The examination of mean globule size and PDI of selected TC-based nanoemulsion formulations was done by using Zetasizer Delsa^TM^ Nano (Backman Coulter, version 2.21). The mean droplet diameter was measured through dynamic light scattering at room temperature. For analysis, the sample (100 µl) dilution was made with distilled water in a 1:100 ratio. The particle diameter was calculated using particle radius and the concentration of nanoemulsion with the lowest nanometre size was used for the studies (24). Deviation in the intensity of scattered light was measured and droplet radius was calculated using Stroke’s equation as follows:

D- kT/6ηR

Where D = Diffusion coefficient, *k* = Boltzmann’s constant, T = Absolute temperature, *η= *viscosity of the medium & R = droplet radius.

*Potential analysis*

The surface charge (potential) of TC-based nanoemulsion formulations was analyzed by light scattering technique through Zetasizer Delsa^TM^ Nano (Backman Coulter, version 2.21). For an experiment, each sample was diluted with distilled water in a 1:100 ratio and analyzed after 24 hr of their preparation. Measuring time was maintained for 60 sec and scattering angle at 90°. The average zeta potential and charges were determined and recorded for all nanoemulsion formulations (25).

*In vitro drug diffusion study*

*In vitro* release from nanoemulsion was analyzed by the membrane diffusion method. TC-based nanoemulsion system (1 ml~ 0.1 mg of TC) was filled in a dialysis bag (molecular cut off 12000 Dalton) which was placed in the release medium (phosphate buffer saline, pH 7.4) at 50 rpm and 37 °C. 1 ml of sample aliquots was taken out from the bags at 0, 0.5, 1, 2, 3, 4, 6, 8, 10, 12, and 24 hr while maintaining sink conditions. All samples were subjected to drug content analysis by UV-spectroscopy at 306 nm. The release of TC from nanoemulsion formulations was compared with the aqueous dispersion. The examination was performed in triplicate and mean values were taken for final calculations (26).

*Transmission electron microscopy (TEM)*

TEM analysis was performed to observe globule morphology and size. Prepared nanoemulsion formulation (F3) was diluted with distilled water in a ratio of 1:100. One drop of the prepared dilution was poured on the film grid of TEM and dried. After drying, the sample was subjected to analysis, and point-to-point estimation of droplets was done by TEM to measure the droplet size of a sample of nanoemulsion (27).

***Preparation of nanoemulgel***

Based on characterization results, the optimized nanoemulsion formulations F2 & F3 were used to make nanoemulgel formulations (NEGs). Each nanoemulsion formulation was incorporated into hydrogel made up of 2% of carbopol gel (carbopol 934 and carbopol 940 in 1:1 ratio). Glycerol and triethanolamine (10%) were also added to stabilize the formulation. The prepared nanoemulgel formulations (NEG2 & NEG3) were kept overnight to swell, and pH was adjusted to 5.8. The prepared NEGs were evaluated for various *in vitro* and *in vivo* parameters (28, 29).

***Characterization of nanoemulgel***

*Appearance and pH determination*

TC-based NEGs were analyzed by physical examination for parameters such as consistency, color, and pH. Accurately weighed quantity (500 mg) of NEGs was diluted with water (10 ml). The pH determined in triplicate for both NEGs was analyzed using a digital pH meter (Mettler Instrument, Germany) (30). 

*Viscosity measurements*

NEGs were subjected to rheological measurement by using a Brookfield Digital Viscometer at 25±0.5 °C. The shear stress of NEGs was examined keeping an increasing to decreasing pressure (15-200-15 Pa) for 60 steps with 10 sec at each step) at 50 rpm (30). 

*Spreadability studies*

The spreadability of NEGs was analyzed by putting the weighed amount of NEG (1 g) beneath a pre-weighed glass plate. The area of spreading for NEG was observed and the result was calculated as a function of an area of spreading to applied mass. The formula for calculating spreadability is as follows (31):

S=(M×L)/T

Where, M= known weight, L= plate length, T= time taken to separate two glass plates. 

*Drug content uniformity*

Accurately weighed quantity of NEGs (500 mg) was diluted with methanol and sonicated for 30 min. After sonication, the extract was centrifuged at 4000 rpm for 15 min. The supernatant layer was removed using a membrane filter (0.45 μm pore size). The dilutions were made with PBS (pH 7.4) and the TC content was analyzed using UV spectroscopy at 306 nm. The examination was performed in triplicate and mean percent values were taken for final calculations (23). 

*Ex-vivo skin permeation studies*

The experiment was performed by using a Franz-diffusion cell with an effective receptor cell volume of 15 ml and diffusion area of 2.545 cm^2^ to analyze percutaneous permeation for the developed nanoemulgel formulation on the shaved skin of rats. The receptor compartment containing phosphate buffer saline (pH 7.4) was maintained at 37±1 °C and kept on constant magnetic stirring at 100 rpm throughout this experiment. The extracted rat skin was kept at room temperature and fixed between donor and receptor compartments by keeping stratum corneum upside and subcutaneous side facing and sticking to receptor compartment. The nanoemulgel formulation (1 ml ~ 0.1 mg of TC) was applied onto the upward side of the skin in the donor cell. The sample aliquots (1 ml) were withdrawn from the receptor chamber with a syringe needle at predetermined time intervals (0, 0.5, 1, 2, 3, 4, 6, 8, 10, 12, and 24 hr) and replaced with the same volume of receptor medium to maintain sink conditions. The skin permeation of optimized nanoemulgel formulations was compared with the plain gel of the drug. The collected aliquots were assessed for drug content by UV spectroscopy at 306 nm. The cumulative amount of drug permeated (CADP) (µg), drug flux (μg/cm^2^ hr), and permeation coefficients were calculated for nanoemulgel and plain gel formulations (32, 33). 

***In vivo studies***

*Ethics and consent to participate*

The animal study was performed in compliance with the guide for care of animals used in the laboratory given by CPCSEA, Government of India. All the experimental procedures used in the study were reviewed by IAEC (Reg. No. 1044/PO/Re/S/07/CPCSEA, 27^th^ Feb 2007 & Project Proposal No. ITS/01/IAEC/2019A). Wistar rats of 200–250 g body weight were obtained from AIIMS, New Delhi, India. The animals were kept in the animal house of I.T.S College of Pharmacy, Ghaziabad, India. The animal groups were allowed for rest at least 1 week before starting testing. Upon taking first treatment, the animal groups were kept in separate hutches in AC room maintained at 22± 2 °C and 60–70 RH% along with meal and water provided *ad libitum* and dark and light cycles of 12 hr each. After the experiment, all rats were euthanized by IP injection of pentobarbital sodium (1 ml/5 kg body weight). The study of wound healing activity against infection caused by *P. aeruginosa *was done using five animals in each Group. The experimental design for *in vivo* activity on animals is given in Table 1. 

*Infected rat burn model*

The experimental model was employed as described by Jacobsen *et al*. (2011) & Stainstraesser *et al*; (2002). After 24 hr of hair removal, the animals were anesthetized with gas anesthesia (2% isoflurane, 38% oxygen, and 60% nitrogen dioxide). Two areas were marked on the back of each animal by immersing them in water (65 °C) for 25 sec. Post washing, the defined areas were dried, marked, and disinfected. 250 µl bacterial solution (1×10^8 ^CFU) of *P. aeruginosa* was applied topically to defined areas. After this, the occlusive dressing was applied immediately to avoid contamination and promote bacterial growth. The bandages and protection clips were applied to rats for stabilization. On day 2 post-infection, animals of the control group were euthanized to observe bacterial counts and considered as a baseline of the overall study period. The rats were treated with equal volume STD and test formulations (n=6 in each group) and the occlusive dressing and bandages were again applied on each animal as mentioned earlier after each application. The study was carried out every day for two weeks. On day 14 of the study, animals from treatment groups were sacrificed and specimens were isolated from the infected wound area aseptically. One specimen of each wound and liver was preserved in paraformaldehyde (4%), stored at 4 °C and used for further histopathological analysis (34, 35).

*Histological assessment*

The biopsy samples were collected from the infected rats from all animal groups on days 0 and 14. The samples were stabilized in 4% buffered paraformaldehyde and subjected to histological analysis using hematoxylin and eosin staining. Samples were analyzed using the following criteria: skin lesions, erythema, scaling, edematous stroma, and hyperplasic epithelium of rat’s skin as well as expansion of hepatic sinusoids, leukocytes, infiltration as well as necrosis of hepatic cells. The entire slot of slides was taken into consideration and each slide was scaled and scored as per above-mentioned criteria (34).

*Statistics model*

One-way ANOVA was used to analyze the *P-*values (significance of difference) for control and test groups (*P*≤0.001). Every test was performed in triplicate and values were provided as average of triplicate values.

***Skin irritancy study***

The skin irritancy test was performed for NEG3 on a healthy albino rat (200 g body weight). NEG3 (1 ml ~ 0.1 mg of drug) was applied to the shaved portion of rat skin. Any change in skin surface before and after NEG3 application was observed at 24, 48, and 72 hr and was compared determining the difference with before application to the skin (36). 

***Stability of nanoemulgel system***

The resistance to phase separation of prepared NEG3 was observed by centrifugation at 4000 rpm for 20 min at ambient temperature. Further, NEG3 was stored for 90 days at temperatures 4 °C and 45 °C along with ambient humidity conditions (35% RH) to observe any phase separation or creaming to check its intrinsic stability. The preparation was checked for drug content, gelling capacity, transparency, and clarity (37).

## Results

***Screening of excipients and solubility studies***

The TC was found to be highly miscible with Labrafac Lipophile (495±4.21 mg/g) and Capryol 90 (305±3.66 mg/g) suggesting the drug’s amphiphilic nature (possessing both hydrophilic and amphiphilic nature) as compared with Captex 355 (highly lipophilic oil phase). Also, TC showed high miscibility with Tween 20 (356±2.11 mg/g) and Transcutol HP (227±2.98 mg/g) (Table 2). 

***Development of pseudo-ternary phase diagram***

**Figure 2.**
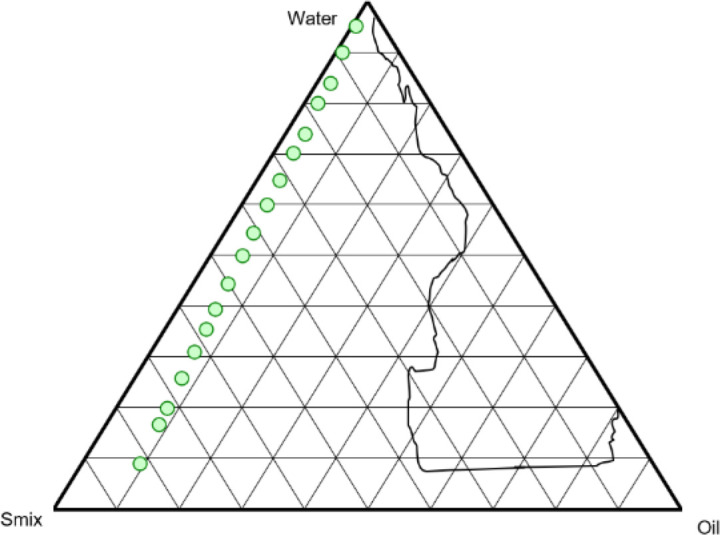
Ternary phase diagram representing the impact of S-CoS mix (in 3:2 ratio) on the nano-emulsion region

The data obtained from solubility studies indicated good solubility of the drug in oil phases Labrafac Lipophile and Capryol 90 and hence the combination of these two oils in a 2:1 ratio was used in experimentation. On other hand, surfactant Tween 20 and co-surfactant Transcutol HP exhibited good solubility for TC and also showed better stability with oil and aqueous phases. To analyze the nanoemulsion region, a pseudo-ternary diagram was plotted using the XL STAT program. The relationship of phase behavior with nanoemulsion system and surfactant-co-surfactant mass ratio (K_m_) can be enumerated via a pseudo-ternary phase diagram (38). The system goes under various transition phases (transparent-translucent-opaque) due to rearrangement of the components within the nanoemulsion system that influences the system’s light-dispersion behavior. In the phase diagram, a region covered by a nanoemulsion system was used to analyze K_m_. Therefore, the larger the area of the nanoemulsion system, the greater will be the nano-emulsification efficiency of the system (39). The surfactant-co-surfactant mixture (S-CoS) and oil phase (Labrafac Lipophile and Capryol 90 in 2:1 ratio) were mixed in 1:1, 2:1, and 3:2 ratio and incorporated in aqueous phase through homogenization method to form nanoemulsion. Since the drug retains a more hydrophobic nature and also the purpose is for topical application, the nanoemulsion (w/o) was formulated. A pseudo-ternary phase diagram with one axis was prepared with oil phase (2:1 ratio), S-CoS mix (3:2 ratio), and aqueous phase highlighting phase behavior, and impact of Km on region acquired by the nanoemulsion system (plotted points) is shown in Figure 2. The concentration of the S-CoS mix is important for barrier formation at the interface as it gets adsorbed at the interface and reduces the energy needed for nanoemulsion formation thus preventing the coalescence among formed nano-globules and increasing the thermodynamic stability of the nanoemulsion formulation. Pseudo-ternary plots indicated that the concentration of oil between 3–5 ml, surfactant between 2–6 ml, and co-surfactant between 1–3 ml was found to promote the nanoemulsion region with needed consistency. 

The phase behavior data of nanoemulsion formulations with different ratios of S-CoS mix demonstrated variation in physical appearance and the size of nano-droplets within the system. For example, Labrafac Lipophile (oil phase) when used with different ratios of S-CoS mix (1:1, 2:1, and 3:2) led to formation of a semi-transparent emulsion. Whereas, the system developed with Capryol 90 (oil phase) with a 1:1 ratio of S-CoS mix resulted in semi-transparent emulsion, and 2:1 and 3:2 ratio of S-CoS mix led to the formation of clear nanoemulsion (>100 nm). The combination of oil phases (Labrafac Lipophile and Capryol 90 in 2:1 ratio) with S-CoS mix (1:1, 2:1, 3:2 ratio) resulted in the formation of clear nanoemulsion with different nano-size range i.e., >100, >50, and <50 nm, respectively. The phase behavior data showed that the oil phase combination with S-CoS (3:2 ratio) exhibited the formation of the lowest size nano-droplets (below 50 nm) within the system. 

***Preparation of nanoemulsion system***

All TC-based nanoemulsion preparations (F1, F2, and F3) were formulated by homogenization process using different ratios of oil medium, surfactant, and co-surfactant. Nanoemulsion (w/o) was prepared with a selected drug (TC) and a total of three nanoemulsion formulations F1, F2, F3 were developed. The optimized composition of drug, oil, surfactant, and co-surfactants is given in Table 3.

***Characterization of nanoemulsion formulations***

The nanoemulsion formulations were subjected to various *in vitro* evaluation parameters. All developed nanoemulsion formulations were completely transparent in appearance and showed no sign of emulsion instability like cracking, coalescence, and creaming. The % drug content in each nanoemulsion formulation (F1-F3) was ranged from 98 to 99%. The mean droplet size, zeta potential, and PDI constitute the main attributes to be examined to achieve stable nanoemulsion formulations. Moreover, these characteristics affect the bulk properties, appearance, product performance, and stability of the nanoemulsion system (40). From table 4, it is observed that increasing the oil concentration from 20 to 40% gives rise to increased mean droplet size while keeping the S-CoS mix concentration (40%) constant (F1>F2>F3). Lower the PDI value represents the higher stability of nanoemulsion formulation. The PDI of the nanoemulsion formulation varied between 0.065±1.14 to 0.691±1.17 following the order F1>F2>F3. Therefore, it confirms that the concentration of oil in formulation composition had a great impact on the mean globule size of nanoemulsions. Zeta potential is a potential difference between the electro-neutral region and surface of the adjacent layer of the nano-droplets and it measures the charge present on the surface of droplets dispersed in nanoemulsion (41). The data of nanoemulsion characterization is given in Table 4.

***Drug diffusion studies***

**Figure 3:**
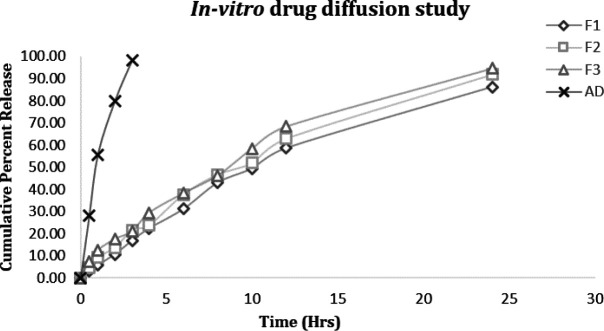
*In vitro* drug diffusion study comparing TC-based nanoemulsion formulations with aqueous dispersions (AD). Nanoemulsion formulations (F1 to F3) show significant *in vitro* release when compared with aqueous dispersion (AD) of the drug over 24 hr (*P*<0.05)

The study was performed using the membrane diffusion method for up to 24 hr (Figure 3). The cumulative percent release of TC from nanoemulsion formulations was compared with aqueous dispersion (AD). The drug release from the nanoemulsion system was found in the range from 86 to 95% up to 24 hr, in a sustained manner (*P*<0.05). The F3 nanoemulsion formulation exhibited maximal release of drug apparently because of the smaller mean droplet size of formulation when compared with other nanoemulsion formulations. On the other hand, the aqueous dispersion showed drug release within the initial hours of the drug release study.

***Transmission electron microscopy (TEM)***

**Figure 4:**
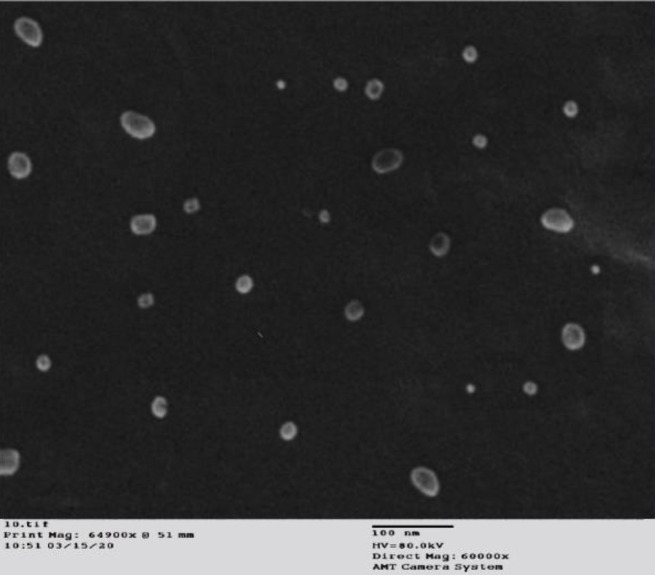
TEM micrograph of thiazole compound based- nanoemulsion formulation (F3)

Based on the characterization results, the F3 nanoemulsion formulation demonstrated the lowest mean size and PDI among all formulations. Also, the zeta potential value of F3 nanoemulsion represents more droplet stability as compared with F1 and F2, as it is reported that zeta potential values more positive than +30 mV and more negative than -30 mV indicate good stability against coalescence (42). Therefore, nanoemulsion formulation F3 was selected for TEM analysis**. **TEM pictures of TC-based nanoemulsion formulation (F3) were captured at 10000 X magnification (Figure 4). TEM study confirmed the presence of nano-sized structures dispersed all over the nanoemulsion system. 

***Preparation of nanoemulgel system***

The nanoemulsion formulations, F2 and F3 were used to make nanoemulgel formulations, NEG2 and NEG3, respectively. The NEGs were assessed for *in vitro* and *in vivo *tests and compared with TC-based plain gel formulation (G1). The plain gel was prepared by adding simply 0.1 mg of TC into 2% carbopol gel by the simple dispersion method. 

***In vitro characterization of nanoemulgel system***

Both prepared NEGs were found to have a milky yellowish-white appearance. The pH of topical preparations must meet the skin pH to avoid skin irritation. The pH of the developed G1, NEG2, and NEG3 was maintained at 5.79±0.06, 5.81±0.02, and 5.80±0.02, respectively. Viscosity measurements for all three formulations, i.e, G1, NEG2, and NEG were performed at ambient temperature (25 °C). Both nanoemulgel formulations (NEG2 and NEG3) exhibited lower viscosity as compared with formulation G1. NEG3 demonstrated the lowest viscosity (79.19±1.71 cp). The result indicated that the viscosity of the NEGs remained the same with increasing shear rate, and NEGs demonstrated Newtonian flow behavior (43). Both nanoemulgel formulations had good consistency and viscosity. Spreadability refers to the maximum surface area on which the formulation spreads over with ease of application. Good spreadability facilitates uniform and easy application of gel preparation applied topically and thus, spreadability plays a crucial role while developing semisolid preparations for topical use (44, 45). The higher the viscosity of formulation the lesser the spreadability it would have. Moreover, spreadability also affects drug distribution and drug penetration throughout the skin. The result showed that the spreading area was increased with applied force due to an increase in the weight for both the developed NEGs formulation and G1 formulation. Thus, no remarkable difference was observed in the spreadability profiles of G1 and NEGs formulations. Drug content was determined for the NEGs and results indicated uniform dispersion of drug (TC) all over the NEG system. Particularly, the % drug content uniformity in NEG2 and NEG3 were found to be 98.65±1.04 and 99.17±1.23, respectively; % drug content was found to be 98.52±0.59 and 99.10±0.62 for NEG2 and NEG3 respectively. The data is shown in Table 5. 

***Ex-vivo skin permeation studies***

**Figure 5:**
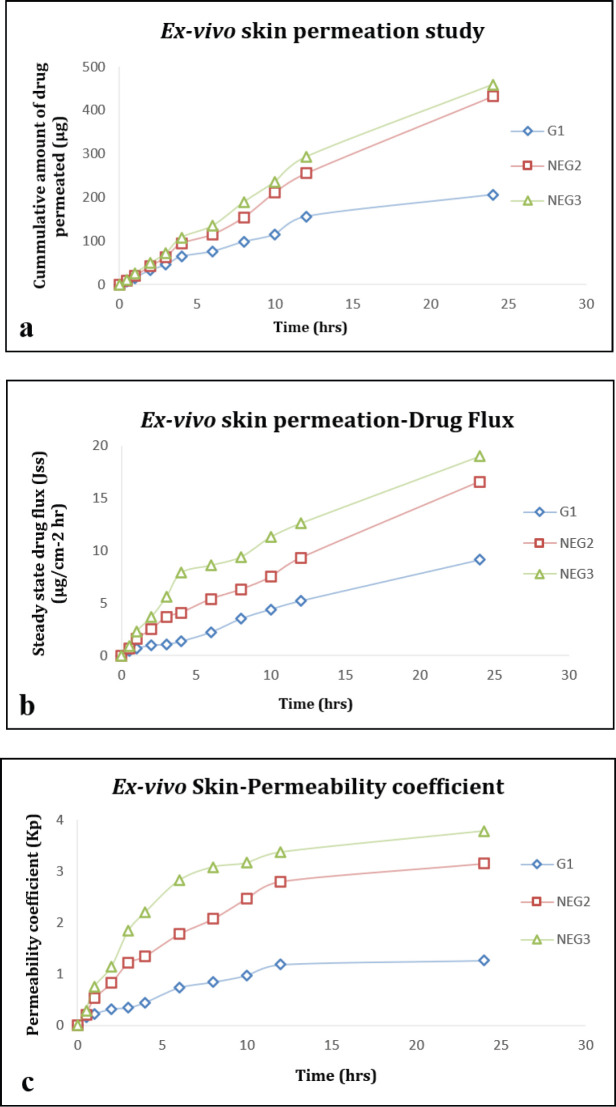
*Ex-vivo *skin permeability comparative study of thiazole compound-based nanoemulgel formulations (NEG2, NEG3) and plain gel (G1).. A) Cumulative % drug release; B) Drug flux; C) Permeability coefficient

A comparative analysis was done with optimized NEGs with G1 using Franz-diffusion cell using excised skin of Wistar rats. Table 5 and Figure 5 (a, b, c) represent the result of the skin permeation study. The cumulative amount of drug permeation was found to be higher in NEG3 as compared with NEG2 whereas G1 exhibited the lowest drug permeation value (Figure 5a). The percutaneous drug flux (Jss) from the nanoemulgel system, NEG3 (18.99±0.76) was more than NEG2 (16.57±0.28) and double the Jss of drug from G1 (9.12±0.52), respectively (Figure 5b). In addition, the permeability coefficient (Kp) of NEG3 (3.78±0.45) was more than NEG2 (3.15±0.03) and double that of G1 (1.26±0.09) (Figure 5c).

***In vivo studies***

The *in vivo* activity of the TC-based nanoemulgel formulations was conducted for 14 days on rats topically infected with *P. aeruginosa*. The parameters like tissue bacterial count, morphological attribute of infected rat burn model, and histological analysis were performed. 

*Tissue bacterial count*

**Figure 6:**
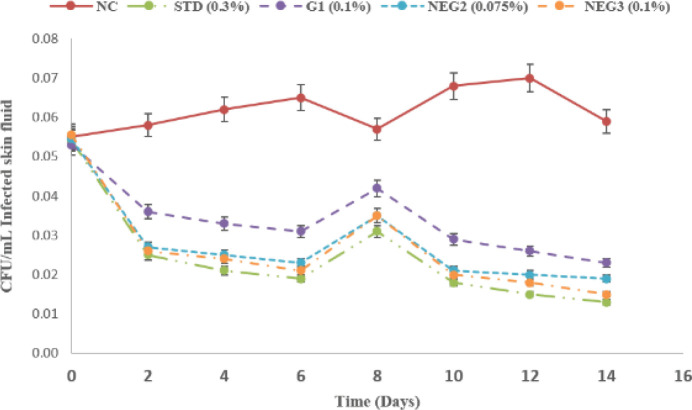
Bacterial count in a *Pseudomonas aeruginosa* infected rat burn model. Effect of topically administered TC and gentamicin against *P. aeruginosa* wound infection. Reduction in the number of CFU was observed in the treatment groups (STD, G1, NEG2, and NEG3) compared with negative control (NC) during the study period. The total bacterial counts within the tissue were found below the infection level (105 CFU/g of tissue) during the study. The detection limit was 102 CFU/g of tissue. The values are shown as mean SEM (*P*≤0.001)

Before starting the treatment, burns showed an infection ranging between 5.8×10^5^ and 7.1×10^5^ CFU/ml infection fluid. Bacterial counts continuously increased in the negative control (NC) group up to a peak at day 12 (1.5×10^7^ CFU/ml infection fluid) during the time course of 14 days. On the other hand, bacterial count in 0.3% gentamicin (STD) and nanoemulgel formulations (NEG2, 0.075%, and NEG3, 0.1%) treated groups were significantly reduced compared with the negative control group throughout the study period (*P*≤0.001). The bacterial count in the group treated with G1 was not significantly reduced (1.4×10^3 ^CFU/ml) compared with standard and nanoemulgel formulations. Nanoemulgel being a lipoidal emulsion-based nanocarrier offers deep skin penetration in the skin layer and demonstrated better biological activity as compared with plain gel. In addition, bacterial count in groups treated with 0.3% gentamicin (2.1×10^2^ CFU/ml) was not significant compared with bacterial counts in groups treated with test-2 (3.1×10^2^ CFU/ml) and test-3 (2.4**×**10^2 ^CFU/ml) formulations. Moreover, the reduction in the bacterial count was observed in a dose-dependent manner in groups treated with NEGs, and NEG3 (0.1% TC) exhibited a better effect than NEG2 (0.075% TC). The statistical data is shown in Table 6 and the comparative effect chart is represented in Figure 6.

*Morphological attribute of infected rat burn model*

**Figure 7:**
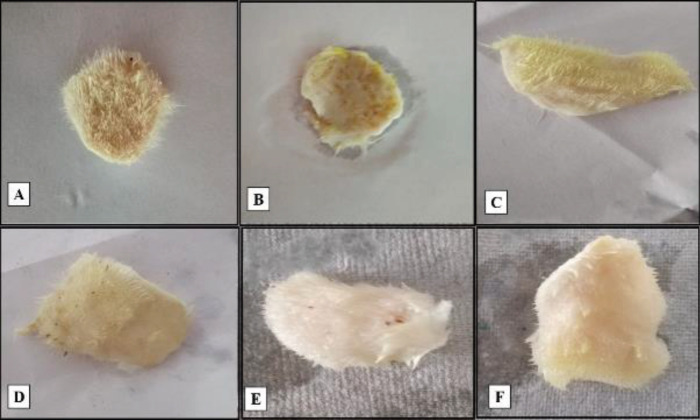
Morphological characteristic of infected rat burn model on day 14. A: Normal morphology in the control group. B: Skin showed indication of erythema, skin exfoliation, and scaling after application of bacterial solution in the NC group. C: Normal morphology after treatment with STD. D: Mild indications of erythema and scaling on the skin in the group treated with G1 (test-1). E, F: No desquamation of skin in groups treated with TC-based NEGs (test-2 and test-3) on day 14

In contrast to normal control, the skin of animals in the NC group showed indications of erythema, skin exfoliating, and scaling after application of a bacterial solution. But almost all signs and symptoms of lesions (desquamation of skin) disappeared on day 14 when the treatment was done with TC-based NEGs (Test 2 and Test 3) and the results were compared with the group treated with STD formulation. Mild indications of skin lesions were observed in the group treated with G1(test-1) indicating the low efficiency of the system (Figure 7).

*Histological assessment*

*i. Skin*

**Figure 8:**
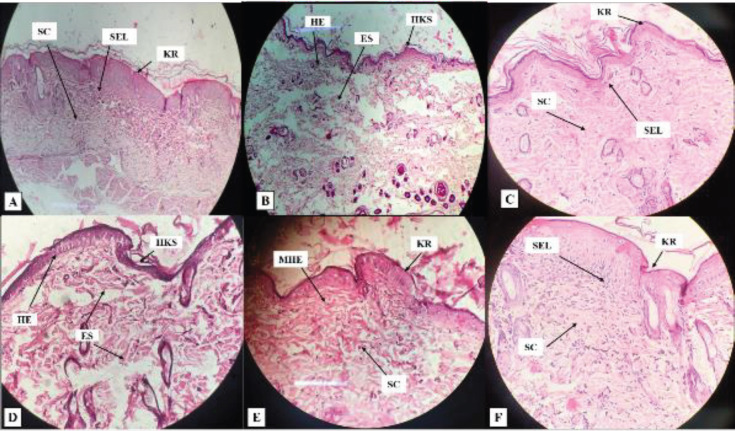
Histopathological analysis of the skin, average values expressed (3 sample ± SEM) in each group. A: Cross-section of normal rat skin shows keratin (KR), Sub-epidermal layer (SEL), and Subcutaneous layer (SC). B: Massive influx of Hyper-keratotic skin (HKS), Hyperplastic epithelium (HE), and Edematous stroma (ES) in the NC group. C: Almost normal skin histology in the group treated with standard drug. D: Hyper-keratotic skin (HKS), Hyperplastic epithelium (HE), and Edematous stroma (ES) in the group treated with G1 (Test-1). E: Normal keratin (KR) and Subcutaneous layer (SC) but Mild hyperplastic epithelium (MHE) in the group treated with NEG-2 (Test 2). F: Normal skin histology in the group treated with NEG-3 (Test 3) on day 14

To analyze the efficacy in *P. aeruginosa *infected and inflamed skin, animal subjects were euthanized on day 14, post-treatment with standard drug, TC-based plain gel (G1), and TC-based nanoemulgel formulations (NEG2 and NEG3). Edematous stroma progressively augmented in all animals applied with bacterial solutions in the model groups. Although G1 treated group skin (picture D) showed the hyperplastic epithelium, test nanoemulgel formulation NEG2 and NEG3 exhibited improved histology as compared with G1. When compared between test nanoemulgel formulations, NEG3 exhibited almost normal histology in animal groups compared with NEG2. The edema and scaling roughly overlapped by that of the test nanoemulgel formulation NEG2 and NEG3 treated groups. The fact here is the higher concentration of TC in NEG3 resulted in better skin wound healing when compared with a control group. This suggests that TC-based nanoemulgel formulations significantly improved the skin condition by preventing pathogenicity when compared with NC group. Besides, the results of the test-3 (NEG3) group were comparable with an STD group as well (Figure 8).

*ii. Liver*

**Figure 9:**
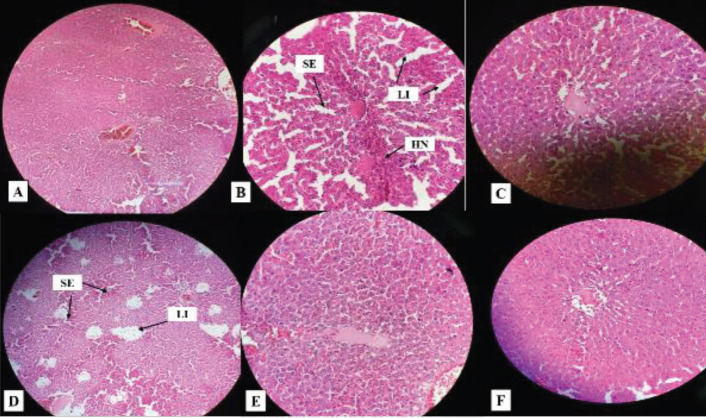
Histopathological analysis of liver of rat. A: Normal histology of hepatocytes shown in the control group. B: Hepatic sinusoid expansion (SE), Leukocytes infiltration (LI), and Hepatocyte's necrosis (HN) in the NC group. C: Almost normal liver histology in the STD group. D: Very mild signs of hepatic sinusoid expansion (SE) and leukocytes infiltration (LI) in the group treated with G1 (test-1). E, F: Both NEG2 (test-2) and NEG3(test-3) represent the almost normal liver histology

Since the ratio of systemic exposure of drugs from topical formulations is very low as compared with oral administration, several reports suggested that drug-induced hepatotoxicity can be possible even upon topical administration (46, 47). Therefore, histological analysis was performed on liver tissue to observe whether the amount of drug that is systemically absorbed does have any adverse effect on the hepatic cells or not upon topical application of nanoemulgel formulation for 14 days. The histology of the liver presented in different groups as follows: Control group rats displayed no changes in liver articulate (A). Expansion of hepatic sinusoids, leukocytes infiltration, as well as necrosis of hepatic cells occurred in the NC group (B). STD group (C) exhibited normal features after treatment. Fascinatingly, rats treated with G1, NEG2, and NEG3 (D, E, and F, respectively) also demonstrated almost normal features of the liver when compared with an NC (untreated) group of animals. The finding suggested that TC does not exert any noticeable major effect on the liver due to its limited systemic exposure. The pictures of histological analysis of all groups are shown in Figure 9. 

***Skin irritancy study***

**Figure 10:**
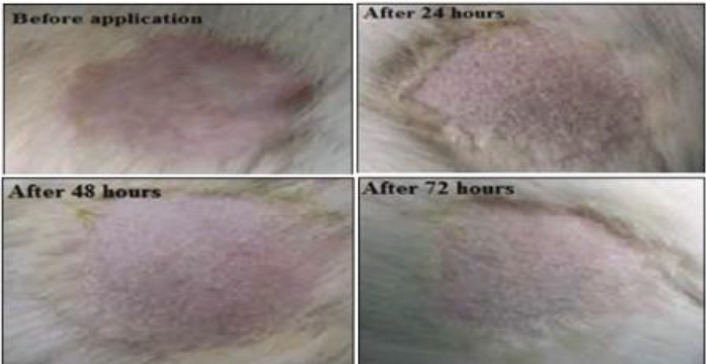
Skin irritancy study showing no sign of inflammation after application of NEG3 on shaved rat skin before application, at days 1, 2, and 3

Based on the results from nanoemulgel characterization, NEG3 (1 g ~ 0.1 mg of TC) was subjected to a skin-irritancy study on the shaved skin of the rat. Following three days of application, NEG3 showed no sign of skin reaction such as redness and inflammation (Figure 10). Hence, it may be considered a suitable delivery system for topical application.

***Stability studies***

The developed NEG3 were found to be stable after being subjected to the centrifugation process for 20 min at 4,000 rpm at room temperature. NEG3 exhibited stability, as creaming and phase separation were not observed when NEG3 was stored at 4 °C and 45 °C with 35% RH (ambient humidity) for 3 months. The formulation was checked for drug content, gelling capacity, pH, transparency, and clarity. Stability analysis of NEG3 indicated its stability, and results are shown in Table 7. 

## Discussion

TC has been reported to have potential antagonistic activity against *P. aeruginosa. *Preformulation studies were performed to select suitable oil and surfactants systems for nanoemulsion preparation. For this, phase behavior analysis was done to observe the influence of different components on the preparation of the nanoemulsion system. The oil phase with remarkable solubility is required to develop a nanoemulsion system that allows the formulation to be loaded with a high drug amount. In addition, the HLB value and specific gravity of the oil system used are major parameters of concern to prepare nanoemulsions via the emulsification process (low energy). The oil system was developed by using a combination of medium-chain triglyceride (Labrafac Lipophile) with medium-chain monoglyceride (Capryol 90) in a ratio of 2:1 to get the oil phase of required HLB with considerable drug loading in nanoemulsion system. The selection of surfactants with optimum HLB value plays an important role in the admixing process of aqueous with oil vehicles during conversion into the nanoemulsion system. The non-ionic surfactants (Polysorbates/Tweens) with high HLB value and low critical micelle concentration (CMS) give rise to more stable globules that are suitable for drug administration. In this study, the suitable non-ionic surfactants named Tween 20 and Transcutol HP (generally considered safe (GRAS grade) were evaluated to be used to develop a TC-based nanoemulsion system. The surfactant Transcutol HP is reported as a suitable co-surfactant for developing a nanoemulsion system for topical application. Thus, Transcutol HP was employed and evaluated in combination with Tween 20 in different ratios. Also, co-surfactant is added to the surfactant system to provide elasticity around the surfactant layer formed over nanoemulsion globules. Besides, co-surfactants importantly overcome the repulsive forces and fluidity of both the aqueous and oil phase, respectively (48). After selection of suitable components, TC-based nanoemulsion formulations were prepared by high-pressure homogenization method and examined to observe product performance and biological efficacy by various *in vitro* characterizations such as mean globule size, PDI, and electro-kinetic (zeta) potential, and drug release studies. The oil concentration remarkably affects the mean globule size of nanoemulsions. The PDI indicating the value 1 or above represent a highly poly-dispersed emulsion. The negative zeta potential values exhibited by nanoemulsion formulations (-24.6±0.46 to -33.65±0.82 mV) may be contributed to several reasons such as the presence of free fatty acids from oil glycerides (Labrafac Lipophile & Capryol 90) used in nanoemulsion formulation, the adsorption of hydroxyl ions at the interface (o/w), the consequent formation of hydrogen bond between hydroxyl ions and ethylene oxide groups of Tween as observed by Liu and coworkers (49). However, the zeta potential (mV) value did not significantly affect by oil and S-CoS mix phases if the drug amount was kept constant. The change in mV value was significantly affected by drug loading concentration in the nanoemulsion preparations. The result indicated that the change in drug concentration is a result of drug dispersion over the surface of oil globules despite being within the globules (43). For topical delivery, ideal droplet size <50 nm and PDI value <1 is preferable as it offers wider surface area and deep penetration of nanoemulsion formulation at the site of application (50). The optimized nanoemulsion formulations (F2 and F3) were incorporated into the hydrogel to make nanoemulgel formulations (NEG2 and NEG3). NEGs exhibited good viscosity, spreadability, and *in vitro* skin permeation studies, as compared with TC-based plain gel. The* in vivo* study revealed that animals treated with NEGs demonstrated pronounced activity against the *P. aeruginosa* induced topical infections as compared with the G1 group. When comparison was seen between both NEGs, NEG3 (0.1% TC) was found to be more effective than NEG2 (0.075% TC) in healing the skin burn infection of *P. aeruginosa* during the entire course of the study (14 days). This was confirmed by histological analysis of rat’s skin indicating a significant reduction in signs like skin lesions, erythema, scaling, edematous stroma, and hyperplasic epithelium in STD and NEGs as compared with NC groups on day 14. Also, histology of the liver demonstrated expansion of hepatic sinusoids, leukocytes, infiltration, as well as necrosis of hepatic cells in the NC group, but groups treated with TC showed no effect on hepatic cells suggesting its low systemic absorption on topical application. Test-3 formulation (NEG3) produced ameliorative action and exhibited significant comparable results when compared with STD (gentamicin treated). NEG3 showed higher permeation and flux values which indicated better retention in the deeper layers of skin, thus prolonging the effect TC at the target site. Skin irritancy studies showed no sign of inflammation or swelling suggesting it is safe for topical application. The stability studies of NEG3 indicated no significant change in product performance after 3 months of storage period and proved its candidature for field utilization. 

**Table 1:** Different animal groups (rats) for *in vivo *studies

**Group**	**Classification**	**Animals (n)**	**Specifications**
I	Control	6	No treatment
II	Negative Control (NC)	6	Bacterial solution of 250 µl (1×10^8 ^CFU) was applied topically to defined areas on the back of animals, incubated, and stabilized for bacterial growth for two days.
III	Standard Control (STD)	6	Animals were treated topically with 1 ml of 0.3% gentamicin marketed topical formulation (STD) for 14 days.
IV	Test I	6	Animals were treated topically with 1 ml of plain gel G1 with 0.1% TC (Test 1) for 14 days.
V	Test II	6	Animals were treated topically with 1 ml of NEG2 with 0.075% TC (Test 2) for 14 days.
VI	Test III	6	Animals were treated topically with 1 ml of NEG3 with 0.1% TC (Test 3) for 14 days.

**Table 2:** Solubility of thiazole compound (TC) in different oils, and surfactants

**Components**	**Solubility (mg/g)**
Labrafac Lipophile	495±4.21
Capryol 90	305±3.66
Captex 355	127±4.95
Tween 20	356±2.11
Transcutol HP	227±2.98
PEG 400	197± 3.89

**Table 3:** Formulation table for nanoemulgel preparations

**Formulations**	**Nano-emulsion composition (%w/w)**				
	**Drug (mg)**	**Oil phase (2:1)**	**Tween 20 (surfactant)**	**Transcutol HP (Co-surfactant)**	**Water (q.s.)**
F1	0.05	40	20	20	20
F2	0.075	30	26.67	13.33	30
F3	0.1	20	24	16	40

**Table 4:** Results of characterization of nano-emulsion system

**Parameters ±SD / formulation code**	**Nano-emulsion formulations**		
	**F1**	**F2**	**F3**
**Appearance**	Transparent	Transparent	Transparent
**Drug content (%)**	97.76±0.84	98.52±0.59	99.10±0.62
**Mean droplet size (nm)**	97.71± 1.37	79.20±1.79	51.04±1.88
**PDI (Mean** **)**	0.691±1.17	0.113±1.36	0.065±1.14
**Zeta Potential (mV)**	-24.6±0.46	-29.4±0.59	-33.65±0.82

*SD= Standard deviation

**Table 5:** Comparative analysis of characterization parameters of plain gel (G1) with nanoemulgel formulations (NEG)

**Parameters ±SD**	**G**_1_	**NEG**_2_	**NEG**_3_
**Appearance**	milky white	milky white	milky white
**pH**	5.79±0.06	5.81±0.02	5.80±0.02
**Viscosity (cp)**	89.4±1.55	82.68±1.63	79.19±1.71
**Spreadability component (cm** ^2^ **/g)**	1.28±0.04	1.36±0.08	1.43±0.05
**Drug content uniformity (mg %)**	97.78±1.13	98.65±1.04	99.17±1.23
**Drug permeated (cumulative) (μg)**	206±11.12	432±13.14	459±10.17
**Drug flux, Jss (μg/cm** ^2^ ** h)**	9.12±0.52	16.57±0.28	18.99±0.76
**Permeation coefficient (Kp×10** ^-3^ **)**	1.26±0.09	3.15±0.06	3.78±0.45

*G1: TC-based plain gel, NEG2 and NEG3:TC-based NEGs, SD= Standard deviation [note: drug flux was calculated from drug permeated (cumulative) vs time (hr). Permeation coefficient (Kp) was calculated by the formula: Kp=Jss/C_0_, where C_0_= initial drug concentration in the donor compartment

**Table 6:** Tissue bacterial count data showing the significance of treatment groups (STD, G1, NEG2, and NEG3) against the NC group

**Time** **(days)**	**NC** **Bacterial count ± SEM**	**STD** **Bacterial count ± SEM**	**G1** **Bacterial count ± SEM**	**NEG2 Bacterial count ± SEM**	**NEG3** **Bacterial count ± SEM**
Basal	7.19 ×10^5^ ±0.23	4.85×10^5^±0.87	6.12×10^5^ ±0.014	5.16 ×10^5^±0.078	5.24×10^5^±0.067
On day 2	1.23 ×10^6 ^±0.06	2.15 ×10^3 ^±0.052***	1.22 ×10^4^±0.065**	3.17 ×10^3 ^±0.017***	1.36 ×10^3 ^±0.026***
On day 4	1.46 ×10^6 ^±0.02	7.27 ×10^2 ^±0.016***	1.08 ×10^4^±0.019*	2.84 ×10^3 ^±0.028***	1.04 ×10^3 ^±0.032***
On day 6	1.63 ×10^6 ^±0.014	5.69 ×10^2^±0.009***	9.14 ×10^3^±0.013*	1.02 ×10^3 ^±0.046**	9.89 ×10^2 ^±0.012***
On day 8	8.27 ×10^5^ ±0.006	6.12 ×10^3 ^±0.013**	7.68 ×10^5^±0.098	7.93 ×10^3 ^±0.046***	9.18 ×10^2 ^±0.012***
On day 10	8.4 ×10^6 ^±0.017	4.35 ×10^2^±0.0057***	5.38 ×10^3^±0.036**	6.19 ×10^2 ^±0.29***	5.71 ×10^2 ^±0.0087***
On day 12	1.5×10^7^ ±0.037	2.18 ×10^2 ^±0.028***	1.23 ×10^3^±0.021*	5.52 ×10^2 ^±0.017***	4.15 ×10^2 ^±0.0057***
On day 14	1.19 ×10^6 ^±0.014	2.1×10^2^ ±0.0057***	1.4×10^3^±0.047*	3.1×10^2^ ±0.027***	2.4×10^2 ^±0.0087

*** (<0.001) - High significant, ** (<0.01) - Moderate significant, * (<0.05) - Low Significant

#Bacterial count (CFU/ml)

#Where NC= Negative control; STD= Standard control; G1= TC-based plain gel; NEG2 & NEG3 = TC-based NEGs; SEM= Standard error of mean (it was calculated by taking the standard deviation and dividing it by the square root of the sample size)

**Table 7:** Stability studies of nanoemulgel formulations

**Storage condition**	**Formulations**	**Drug content (%)**	**Gelling capacity**	**pH**	**Transparency**	**Clarity**
**4± 1** ^o^ **C, Ambient humidity**	NEG3	97.41±0.36	+++	5.79±0.03	++	Clear
**40±1** ^o^ **C,** **Ambient humidity**	NEG3	96.81±2.73	+++	5.81±0.05	++	Clear

## Conclusion

Based on the findings, the study suggests TC-based NEG is a promising system to treat *P. aeruginosa* induced surface-infection. However, this work is only limited to treating skin infections and can be further investigated to find out the TC effect either alone or in combination with other potent antibiotics to synergize the overall effect against systemic infections caused by *P. aeruginosa*. 

## Authors’ Contributions

The original idea was uprooted by Snigdha Bhardwaj. The research work was carried out by Snigdha Bhardwaj and guided by Dr. Sonam Bhatia, Dr Pushpraj S. Gupta, and Dr Shaminder Singh. All the data/results analysis, drafting, and proofreading of the manuscript are thoroughly done by Dr. Sonam Bhatia and Dr. Pushpraj S. Gupta. All authors have read and approved the manuscript.

## Conflicts of Interest

All authors declare that they have no conflicts of interest.

## References

[1] Kon K, Rai M, 1st ed (p). Antibiotic resistance mechanisms and new antimicrobial approaches. Academic Press Elsevier Inc;USA.

[2] Gomez MI, Prince A (2007). Opportunistic infections in lung disease: pseudomonas infections in cystic fibrosis. Curr Opin Pharmacol.

[3] Maura D, Ballok AE, Rahme LG (2016). Considerations and caveats in anti-virulence drug development. Curr Opin Microbiol.

[4] Wagner S, Sommer R, Hinsberger S, Lu C, Hartmann RW, Empting M (2016). Novel strategies for the treatment of Pseudomonas aeruginosa infections. J Med Chem.

[5] Clatworthy AE, Pierson E, Hung DT (2007). Targeting virulence: a new paradigm for antimicrobial therapy. Nat Chem Biol.

[6] Kariminik A, Majid BS, Kheirkhah B (2017). Pseudomonas aeruginosa quorum sensing modulates immune responses: An updated review article. Immunol Lett.

[7] Bhardwaj S, Bhatia S, Singh S, Franco Jr F (2021). Growing emergence of drug-resistant Pseudomonas aeruginosa and attenuation of its virulence using quorum sensing inhibitors: A critical review. Iran J Basic Med Sci.

[8] Chourasiya SS, Kathuria D, Singh S, Sonawane VC, Chakrabortia AK, Bharatam PV (2015). Design, synthesis and biological evaluation of novel unsymmetrical azines as quorum sensing inhibitors. RSC Adv.

[9] Bhardwaj S, Tiwari A (2021). Nanoemulgel: A promising nanolipoidal-emulsion based drug delivery system in managing psoriasis. Dhaka Univ J Pharm Sci.

[10] Tyagi A, Bhardwaj S, Kumar Gaur P, Singh M (2021). A review on topical nanocarrier system of nsaids in the management of soft tissue injury. Int J All Res Educ Sci Methods.

[11] Tiwari A, Bhardwaj S, Kumar Gaur P, Singh AP, Barman M (2020). Potential advancement of nanocarriers in topical drug delivery: A mini review. Lett Appl NanobioSci.

[12] Ali SS, Bhardwaj S, Khan NA, Imam SS, Kala C ( 2021). Phytoconstituents loaded nanomedicines for arthritis management. inflammatory biomarkers as Targeted Herbal drug discovery tool: A pharmacological approach to nanomedicines published by apple academic press.

[13] Bhardwaj S, Bhatia S (2020). Development and characterization of niosomal gel system using lallementia royaleana benth  mucilage for the treatment of rheumatoid arthritis. Iran J Pharm Res.

[14] Bhardwaj S, Gaur PK, Tiwari A (2022). Development of topical nanoemulgel using combined therapy for treating psoriasis. Assay Drug Dev Technol.

[15] Basera K, Bhatt G, Kothiyal P, Gupta P (2015). Nanoemulgel: A novel formulation approach for topical deliverey of hydrophobic drugs. World J Pharm Pharm Sci.

[16] Tamjidi F, Nasirpour A, Varshosaz J, Shahedi M (2013). Nanostructured lipid carriers (NLC): A potential delivery system for bioactive food molecules. Innov Food Sci Emerg Technol.

[17] Shakeel F, Baboota S, Ahuja A, Ali J, Aqil M, Shafiq S (2007). Nanoemulsions as vehicles for transdermal delivery of aceclofenac. AAPS Pharm Sci Tech.

[18] Shafiq-un-Nabi S, Shakeel F, Talegaonkar S, Ali J, Baboota S, Ahuja A (2007). Formulation development and optimization using nanoemulsion technique: a technical note. AAPS Pharm Sci Tech.

[19] Ahmad J, Kohli K, Mir SR, Amin S (2011). Formulation of self-nanoemulsifying drug delivery system for telmisartan with improved dissolution and oral bioavailability. J Dispers Sci Technol.

[20] Ahmad J, Mir SR, Kohli K, Amin S (2014). Effect of oil and co-surfactant on the formation of solutol hs 15 based colloidal drug carrier by box-behnken statistical design. Colloids Surf Physicochem Eng Asp.

[21] Algahtani MS, Ahmad MZ, Ahmad J (2020). Nanoemulgel for improved topical delivery of retinyl palmitate: formulation design and stability evaluation. Nanomaterials (Basel).

[22] Bouchemal K, S Briançon, Perrier E, Fessi H (2004). Nano-emulsion formulation using spontaneous emulsification: solvent, oil and surfactant optimization. Int J Pharm.

[23] Amin S, Sarfenejad A, Ahmad J, Kohli K, Mir SR (2013). Nanovesicular transfersomes for enhanced systemic delivery of telmisartan. Adv Sci Eng Med.

[24] Tadros T, Izquierdo P, Esquena J, Solans C (2004). Formation and stability of nano-emulsions. Adv Colloid Interface Sci.

[25] Shafiq S, Shakeel F, Talegaonkar S, Ahmad FJ, Khar RK, Ali M (2007). Development and bioavailability assessment of ramipril nanoemulsion formulation. Eur J Pharm Biopharm.

[26] Nava G, Pinon E, Mendoza L, Mendoza N, Quintanar D, Ganem A (2011). Formulation and in vitro, ex vivo and in vitro evaluation of elastic liposomes for transdermal delivery of ketorolac tromethamine. Pharmaceutics.

[27] Bhattacharya S, Prajapati BG (2017). Formulation and optimization of celecoxib nanoemulgel. Asian J Pharm Clin Res.

[28] Verma S, Singh AK, Mukerjee A (2016). Formulation & evaluation of ketoconazole nanoemulgel. World J Pharm Pharm Sci.

[29] Ghosh V, Saranya S, Mukherjee A, Chandrasekaran N (2013). Cinnamon oil nanoemulsion formulation by ultrasonic emulsification: investigation of its bactericidal activity. J Nanosci Nanotechnol.

[30] Ahmad J, Gautam A, Komath S, Bano M, Garg A, Jain K (2019). Topical nano-emulgel for skin disorders: Formulation approach and characterization. Recent Pat Antiinfect Drug Discov.

[31] Karade PG, Rohit RS, Chougale DD, Bhise SB (2012). Formulation and evaluation of celecoxib gel. J Drug Deliv Ther.

[32] Okyar A, Yildiz A, Aksu B, Çınar C, Ozsoy Y, Baktır G (2008). Effect of terpenes as penetration enhancers on percutaneous penetration of tiaprofenic acid through pig skin. Acta Pharm Sci.

[33] Plessis J, Egbaria K, Weiner N (1992). Influence of formulation factors on the deposition of liposomal components into the different strata of the skin. J Soc Cosmet Chem.

[34] Jacobsen F, Fisahn C, Sorkin M, Thiele I, Hirsch T, Stricker I (2011). Efficacy of topically delivered moxifloxacin against wound infection by Pseudomonas aeruginosa and methicillin-resistant Staphylococcus aureus. Antimicrob Agents Chemother.

[35] Steinstraesser L, Tack BF, Waring AJ, Hong T, Boo LM, Fan MH (2002). Activity of novispirin G10 against Pseudomonas aeruginosa in vitro and in infected burns. Antimicrob Agents Chemother.

[36] Sintov A, Zeevi A, Uzan R, Nyska A (1999). Influence of pharmaceutical gel vehicles containing oleic acid/sodium oleate combinations on hairless mouse skin, a histological evaluation. Eur J Pharm Biopharm.

[37] Iradhati AH, Jufri M (2017). Formulation and physical stability test of griseofulvin microemulsion gel. Int J Appl Pharm.

[38] Akhter S, Anwar M, Siddiqui MA, Ahmad I, Ahmad J, Ahmad MZ (2016). Improving the topical ocular pharmacokinetics of an immunosuppressant agent with mucoadhesive nanoemulsions: formulation development, in vitro and in vivo studies. Colloids Surf B Biointerfaces.

[39] Su R, Yang L, Wang Y, Yu S, Guo Y, Deng J (2017). Formulation, development, and optimization of a novel octyldodecanol-based nanoemulsion for transdermal delivery of ceramide IIIB. Int J Nanomed.

[40] Sinko PJ, Singh Y (2011). Martin’s physical pharmacy and pharmaceutical sciences: physical chemical and biopharmaceutical principles in the pharmaceutical sciences; walter kluer: Alphen aan den rijn, south holland, The Netherlands.

[41] Danaei M, Dehghankhold M, Ataei S, Hasanzadeh Davarani F, Javanmard R, Dokhani A (2018). Impact of particle size and polydispersity index on the clinical applications of lipidic nanocarrier systems. Pharmaceutics.

[42] Kadu PJ, Kushare SS, Thacker DD, Gattani, SG (2011). Enhancement of oral bioavailability of atorvastatin calcium by self-emulsifying drug delivery systems (SEDDS). Pharm Dev Technol.

[43] Eskandar NG, Simovic S, Prestidge CA (2009). Chemical stability and phase distribution of all-trans-retinol in nanoparticle-coated emulsions. Int J Pharm.

[44] Dantas MG, Reis SA, Damasceno CM, Rolim LA, Rolim-Neto PJ, Carvalho FO, Quintans-Junior LJ, Almeida JR (2016). Development and evaluation of stability of a gel formulation containing the monoterpene borneol. Sci World J.

[45] Chen MX, Alexander KS, Baki G (2016). Formulation and evaluation of antibacterial creams and gels containing metal ions for topical application. J Pharm (Cairo).

[46] Kienzler JL, Gold M, Nollevaux F (2010). Systemic bioavailability of topical diclofenac sodium gel 1% versus oral diclofenac sodium in healthy volunteers. J Clin Pharmacol.

[47] LiverTox: Clinical and research information on drug-induced liver injury [Internet]. Bethesda (MD): National Institute of Diabetes and Digestive and Kidney Diseases; 2012. Diclofenac.

[48] Dizaj SM (2013). Preparation and study of vitamin A palmitate microemulsion drug delivery system and investigation of co-surfactant effect. J Nanostruct Chem.

[49] Liu W, Sun D (2006). Formation and stability of paraffin oil-in-water nano-emulsions prepared by the emulsion inversion point method. J Colloid Interface Sci.

[50] Campos FF, Calpena Campmany AC, Delgado GR, Serrano OL, Naveros BC (2012). Development and characterization of a novel nystatin-loaded nanoemulsion for the buccal treatment of candidosis: Ultrastructural effects and release studies. J Pharm Sci.

